# yuDetecting the percent of peripheral blood mononuclear cells displaying p-STAT-3 in malignant glioma patients

**DOI:** 10.1186/1479-5876-7-92

**Published:** 2009-11-09

**Authors:** William Humphries, Yongtao Wang, Wei Qiao, Chantal Reina-Ortiz, Mohamed K Abou-Ghazal, Lamonne M Crutcher, Jun Wei, Ling-Yuan Kong, Raymond Sawaya, Ganesh Rao, Jeffrey Weinberg, Sujit S Prabhu, Gregory N Fuller, Amy B Heimberger

**Affiliations:** 1 Department of Neurosurgery, The University of Texas M. D. Anderson Cancer Center, Houston, TX, USA; 2 Department of Biostatistics, The University of Texas M. D. Anderson Cancer Center, Houston, TX, USA; 3 Department of Pathology, The University of Texas M. D. Anderson Cancer Center, Houston, TX, USA

## Abstract

**Background:**

The signal transducer and activator of transcription 3 (STAT-3) is frequently overexpressed in cancer cells, propagates tumorigenesis, and is a key regulator of immune suppression in cancer patients. The presence of phosphorylated STAT-3 (p-STAT-3) in the tumor can induce p-STAT-3 in tumor-associated immune cells that can return to the circulatory system. We hypothesized that the number of peripheral blood mononuclear cells (PBMCs) displaying p-STAT-3 would be increased in glioma patients, which would correlate with the extent of tumor-expressed p-STAT-3, and that higher p-STAT-3 levels in peripheral blood would correlate with a higher fraction of immune-suppressive regulatory T cells (Tregs).

**Methods:**

We measured the percentage of PBMCs displaying p-STAT-3 in 19 healthy donors and 45 patients with primary brain tumors. The level of p-STAT-3 in tumor tissue was determined by immunohistochemistry. The degree of immune suppression was determined based on the fraction of Tregs in the CD4 compartment.

**Results:**

Healthy donors had 4.8 ± 3.6% of PBMCs that expressed p-STAT-3, while the mean proportion of PBMCs displaying p-STAT-3 in patients with GBM was 11.8 ± 13.5% (*P *= 0.03). We did not observe a correlation by Spearman correlation between the degree of p-STAT-3 levels in the tumor and the percent of PBMCs displaying p-STAT-3. Furthermore, the percent of PBMCs displaying p-STAT-3 in glioma patients was not directly correlated with the fraction of Tregs in the CD4 compartment.

**Conclusion:**

We conclude that the percent of PBMCs displaying p-STAT-3 may be increased in malignant glioma patients.

## Background

Malignant brain tumors have the capability to evade immune surveillance and impede antitumor immune responses, which may lead to continued growth and increased malignancy. In many malignancies, the signal transducer and activator of transcription 3 (STAT-3) plays an integral role in modulating oncogenesis, inhibiting apoptosis, and suppressing immunity [1,2]. STAT-3 has been found to be constitutively activated in 50-90% of all malignant tumors, including 53% of anaplastic astrocytomas and 53% of glioblastomas [3]. In gliomas, cytokines, such as interleukin (IL)-6 (IL-6) [4,5] and epidermal growth factor [5], can cause subsequent phosphorylation and activation of STAT-3. The phosphorylated STAT-3 (p-STAT-3) then translocates into the nucleus and induces a variety of transcriptional factors that propagate tumorigenesis [1] and up-regulate tumor-mediated immunosuppressive factors [2]. These factors include IL-10 [6,7] that adversely influences Th1-mediated cytotoxic immune responses at multiple levels and is essential for regulatory T cells (Tregs) function [8,9], vascular endothelial growth factor [10] that inhibits dendritic cell maturation and activation by inhibiting co-stimulatory molecule expression [11], PGE_2 _[12] that induces the immune suppressive Th17 cell [13], and TGF-β [14] that induces Tregs, inhibits T cell proliferation and down-modulates the IL-2 receptor. These STAT-3-regulated tumor secreted factors then activate STAT-3 in diverse immune cells [15] including macrophages and monocytes [16-18], dendritic cells [2], T cells [19], and Tregs [20]. More specifically, IL-2 has been shown to regulate *FoxP3 *expression in human CD4^+^CD25^+ ^Tregs by inducing STAT-3 binding of the first intron of the FoxP3 gene [20]. Because STAT-3 target genes encode many factors that activate STAT-3 in the immune cells, possibly a feed-forward mechanism for activation of STAT-3 in both the tumor cells and the immune cells within the tumor microenvironment is initiated as proposed by Kortylewski [21]. The cumulative response of activating the STAT3 pathway in the immune system is anti-inflammatory by a combination of suppressing macrophage activation [22,23], reducing the cellular cytotoxicity of natural killer cells and neutrophils, reducing the expression of major histocompatibility complex (MHC) II, CD80, CD86, and IL-12 in dendritic cells rendering them unable to stimulate T cells and generate antitumor immunity [15] and enhancing Treg activity [20]. Within the immune cells, γ-IFN has been shown to be down-regulated by p-STAT-3 [15] and accordingly γ-IFN levels have been shown to be decreased in glioma patient PBMCs [24]. The ablation of STAT-3 activity in only the immune cells results in marked antitumor effects *in vivo*, indicating that STAT-3 expression within the immune cells is what restrains antitumor eradication [15]. Furthermore, we have shown that p-STAT-3 blockade in immune cells restores immune responses [25] and inhibits Treg induction [26]. Overall, p-STAT-3 regulates immune suppression and tumor progression via multiple redundant mechanisms [18,22,23,27,28].

Primed CD8^+ ^cytotoxic T cells have been shown to gain central nervous system (CNS) access [29,30], and immune cells are present in tumors and the surrounding brain parenchyma [30]. These immune cells may then traffic outside the CNS [31,32] by following the lymphatic drainage through the brain via the Virchow-Robin spaces to lymphatics beneath the cribriform plate, ultimately reaching the cervical lymph nodes [33,34]. Thus, CNS tumor-elaborated substances are capable of reaching the immune system and peripheral blood stream. Therefore, we hypothesized that the percent of peripheral blood mononuclear cells (PBMCs) displaying p-STAT-3 may be increased in malignant glioma patients. The p-STAT-3 levels may be increased in the peripheral blood in two ways: (1) a tumor with p-STAT-3 would subsequently induce p-STAT-3 in tumor-associated immune cells, which would then reenter systemic circulation or (2) p-STAT-3 transcriptional induced tumor-secreted products could induce p-STAT-3 in immune cells in the cervical lymph nodes, which then are detected in the peripheral circulation. Therefore, we measured p-STAT-3 in glioma patients' peripheral blood mononuclear cells (PBMCs) and compared these levels to those of healthy donors. We also tested the hypothesis that the level of p-STAT-3 in a tumor would correlate with the percent of PBMCs displaying p-STAT-3. To evaluate whether the percent of PBMCs displaying p-STAT-3 correlated with immune suppression, we tested for a correlation between the percent of PBMCs displaying p-STAT-3 and the fraction of enhanced Tregs in the systemic circulation [35] especially since p-STAT-3 binds to the first intron of the FoxP3 gene [20] and because STAT-3 inhibitors have been shown to inhibit Tregs [26,36].

## Materials and methods

### Acquisition of peripheral blood and tumor specimens

Peripheral blood samples (*N *= 45) were collected from patients prospectively, usually intraoperatively before skin incision and after the administration of 10 mg of dexamethasone, or during a routine clinic visit during a 1 year time period (3/3/08-2/18/09). Eligible participants included any glioma patients undergoing surgical resection or treatment at The University of Texas M. D. Anderson Cancer Center and their tumor pathology was characterized by a neuropathologist at The University of Texas M. D. Anderson Cancer Center according to the 2007 criteria of the World Health Organization (WHO) [37]. This study was conducted under protocol # LAB03-0687, which was approved by the institutional review board of M. D. Anderson Cancer Center, and informed consent was obtained. Since M. D. Anderson Cancer Center is focused exclusively on the oncological patient population, controls of non-oncological patients undergoing surgery are not routinely available. The normal, healthy volunteers did not undergo surgical procedures but their blood was collected and transported in an identical manner compared to the surgical patients.

### Isolation of PBMCs and staining for p-STAT-3

Approximately 30-40 mL of peripheral blood was collected in sodium heparin tubes (BD Vacutainer, Becton Dickinson, Franklin Lakes, NJ) and transported on ice to our laboratory. Blood samples were then subjected to density gradient centrifugation using Ficoll-Paque (Amersham Biosciences, Piscataway, NJ). PBMCs were isolated and washed twice in sterile phosphate-buffered saline (PBS) solution at 1700 rpm for 5 min. After washing, 20 × 10^6 ^cells were resuspended in 0.5 mL of PBS. Paraformaldehyde (0.5 mL), prewarmed to 37°C, was added to achieve a final concentration of 2%, and the solution was incubated for 10 min at 37°C and then chilled on ice for 1 min. Next, 5 × 10^6 ^cells were transferred into 4 separate wells of a 96-well U-bottomed plate (Corning Incorporated, Lowell, MA). To make the cells permeable, we removed the paraformaldehyde by pelleting the cells at 1500 rpm for 5 min, resuspending them in prechilled 90% methanol, and incubating them on ice for 30 min. The cells were then pelleted at 1500 rpm in fluorescence-activated cell sorter (FACS) buffer (PBS with 0.5% bovine serum albumin) for 2.5 min at 1500 rpm. The cells were resuspended in 45 μL of FACS buffer/well, and 5 μL of mouse phycoerythrin (PE)-labeled antihuman p-STAT-3 (Y705) antibody (BD Biosciences, San Jose, CA) was added. Matched control wells included 5 μL of PE-labeled IgG2a-κ isotype control (eBioscience, San Diego, CA). The cells were incubated for 60 min at room temperature and washed with 200 μL of FACS buffer/well for 2.5 min at 1500 rpm. The cells were then resuspended in 250 μL of FACS buffer/well and transferred to FACS tubes for flow cytometry (FACSCalibur; BD Biosciences). Duplicate specimens were parallel processed in most cases, but insufficient collection of intraoperative blood sometimes precluded this analysis.

### Determination of Tregs in peripheral blood of patients withgliomas

For subset analysis, after we became proficient at analyzing PBMC p-STAT-3, after the isolation of PBMCs as described above, additional aliquots of approximately 2.5 × 10^6 ^cells were plated into duplicate wells of 96-well V-bottomed plates. The cells were then centrifuged at 1500 rpm for 2.5 min, after which they were washed twice with FACS buffer at 1500 rpm for 2.5 min. Surface staining was done using 5 μL of FITC--labeled antihuman CD4 (Pharmingen, San Diego, CA) in 45 μL of FACS buffer and 5 μL of APC-labeled antihuman CD25 (Pharmingen) for 15 min at 4°C. Cells were then washed with FACS buffer and permeabilized with 1:3 Cytofix/Cytoperm (eBioscience) for 2 h at 4°C. The cells were then centrifuged at 1500 rpm for 2.5 min and washed once with FACS buffer and 3 times with 1:3 PermWash (eBioscience). The cells were stained intracellularly with 5 μL PE-antihuman FoxP3 antibody (eBioscience) diluted in 45 μL PermWash for 30 min at 4°C. For an isotype control, 5 μL PE-antimouse IgG antibody (eBioscience) diluted in 45 μL PermWash was added to matched wells. Cells were washed with 200 μL PermWash (BD Biosciences) and then with 200 μL FACS buffer, and then were transferred into FACS tubes for flow cytometry analysis. The calculated Treg fraction was designated as the number of CD4^+^CD25^+^FoxP3^+ ^cells divided by the total CD4^+ ^population.

### Immunohistochemical analysis of p-STAT-3 in gliomas

After formalin-fixed, paraffin-embedded sections of the gliomas were deparaffinized in xylene, they were rehydrated in ethanol. Endogenous peroxidase was blocked with 0.3% hydrogen peroxide/methanol for 10 min at room temperature before antigen retrieval was begun. Antigen retrieval for p-STAT-3 consisted of immersing the sections in a citrate-buffered solution (pH 6.0) and heating them in a microwave oven for 20 min. The sections were then cooled to room temperature for 40 min. After blocking with a protein-block serum-free solution (DAKO, Carpinteria, CA), anti-p-STAT-3 (tyrosine^705^) antibody (1:50; Cell Signaling Technology, Danvers, MA, that recognizes the same epitope as Y705) was added, and specimens were incubated overnight in a humidified box at 4°C. Slides were secondarily stained with biotin-labeled secondary antibody (biotinylated link universal solution) (DAKO) for 60 min at room temperature. Finally, streptavidin-horseradish peroxidase (DAKO) was added, and slides were incubated for 30 min at room temperature. Diaminobenzidine (DAKO) was used as the chromogen, and color development was stopped by gently dipping slides into distilled water. The nuclei were then counterstained with hematoxylin. A glioma tissue microarray [3] served as a positive control for p-STAT-3 staining. The negative control was created by omitting the primary antibody from the immunohistochemical analysis and replacing it with the protein-block serum-free solution.

Three independent observers (WH, YW, GNF) quantitatively evaluated p-STAT-3 by analyzing the core of each specimen using high-power fields (maximum: × 40 objective and × 10 eyepiece, Axioskop 40, Carl Zeiss, Inc). Each observer recorded the absolute number of tumor cells staining positive for nuclear p-STAT-3 per × 200 high-power field. The endothelial cells and infiltrating immune cells displaying p-STAT-3 were not included in this number. If there were discrepancies between observers' recorded numbers, the observers recounted the number of positively stained cells in each specimen; if they still disagreed, the neuropathologist (GNF) conducted the final arbitration.

### Statistical analysis

For each specimen, we attempted to analyze duplicate samples to measure the percentage of PBMCs displaying p-STAT-3. The sample size is denoted as *N *and the number of measurements is represented as *n*. Mixed models were used to compare differences in the percent of PBMCs displaying p-STAT-3 between patients with glioma and healthy donors. In this way, the correlation between the two samples from each subject was taken into consideration. The Spearman correlation was used to analyze the association between the Treg fraction and the scatter plot with Loess smooth curves were presented to demonstrate the relationship. Comparison of Treg fraction difference between normal and tumor patients was conducted using Wilcoxon tests. All computations were carried out in SAS software (version 9.1; SAS Institute, Cary, NC) and SPLUS software (version 8.0.;TIBCO, Palo Alto, CA). Values at which differences were considered statistically significant were *P *< 0.05.

## Results

### Study population

This study included blood samples from 45 patients with gliomas who were treated at M. D. Anderson. Table 1 summarizes the overall composition of the study group and includes characteristics of the cohort, including age, gender, Karnofsky performance status score, and pathologic diagnosis. The glioblastoma multiforme (GBM) cases were further characterized according to whether the glioma was newly diagnosed, recurrent, or without tumor progression. The GBM patients without tumor progression on MRI consisted of two patients undergoing treatment with temozolomide and immunotherapy that were at least six months from their initial surgery and two patients undergoing surgical debridement for infection. One GBM patient undergoing stereotactic biopsy for determination of radiation necrosis was placed in the GBM without tumor progression group. The mean age for the healthy donors was 44 ± 12.8 and 47% were male.

**Table 1:** Patient characteristics across different tumor types

**Pathology**	**WHO grade**		**Age**	**KPS**	**Gender**			
		**Mean**	**Median (min, max)**	**Median (min, max)**	**Female**		**Male**	
					
					**N**	**%**	**N**	**%**

Ganglioglioma	II	34.7 ± 3.6	34.7 (32.1, 37.2)	100 (100, 100)	1	50.00	1	50.0

AA/AO	III	47.3 ± 8.6	49.4 (29.3, 56.8)	90 (90, 100)	3	30.00	7	70.0

New GBM	IV	57.2 ± 12.8	57.4 (26.4, 77.0)	90 (60, 100)	7	46.6	8	53.3

Not Progressing GBM	IV	54.2 ± 12.9	61.5 (39.3, 61.8)	100 (90, 100)	1	20.0	4	80.0

Recurrent GBM	IV	46.4 ± 19.4	47.1 (20.6, 68.9)	80 (50, 100)	4	30.8	9	62.2

WHO -- World Health Organization; KPS -- Karnofsky Performance Status

### Determination of p-STAT-3 in PBMCs of glioma patients

**Figure 1:**
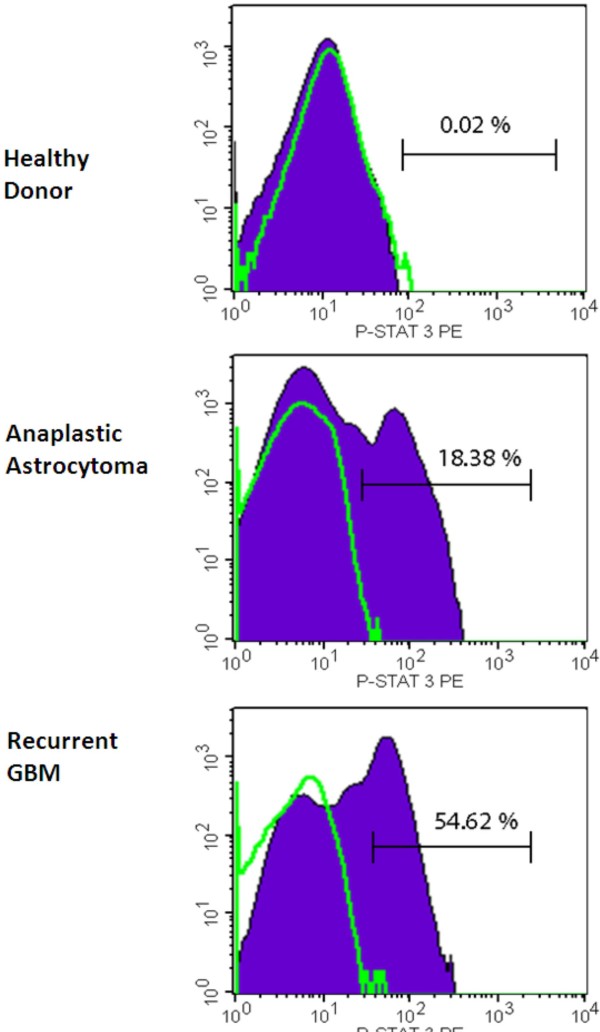
**Representative examples of PBMCs isolated from blood samples obtained from a healthy donor, a patient with an anaplastic astrocytoma with positive p-STAT-3 expression, and a patient with a recurrent GBM with positive p-STAT-3 expression**. The samples were fixed in paraformaldehyde, permeabilized, stained with mouse PE-labeled antihuman p-STAT-3 (Y705) antibody, and analyzed by FACS. The isotype control is in green.

Representative positive specimens are shown in Fig. 1. Sequential measurements of the same sample over time demonstrated a loss of p-STAT-3 in fresh specimens after 24 h (data not shown) and in frozen specimens, indicating samples should be processed and analyzed as soon as possible after being collected from the patient. The MFI of p-STAT-3 among samples was similar.

### Higher percentage of PBMCs expresses p-STAT-3 in glioma patients than in healthy donors

**Figure 2:**
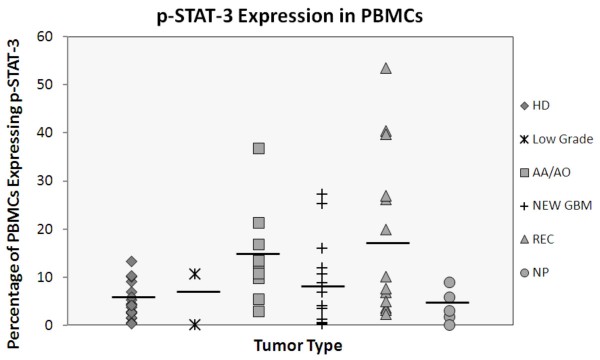
**Expression of p-STAT-3 is enhanced in PBMCs from glioma patients**. PBMCs were isolated from blood samples obtained from healthy donors (*N *= 19) and glioma patients (*N *= 45). The samples were intracellularly stained with antihuman p-STAT-3 and analyzed by FACS. The percentage of p-STAT-3-positive PBMCs differed significantly between healthy donors and glioma patients. Abbreviations used: Anaplastic astrocytoma, AA; Anaplastic oligodendroglioma, AO; Glioblastoma multiforme, GBM; Normal, healthy donor, HD; Recurrent, REC; No progression, NP.

The mean percentage of PBMCs displaying p-STAT-3 from all healthy donors (denoted by the diamonds) (*N *= 19; *n *= 38) was 4.8 ± 3.6%. In all GBM patients (*N *= 33; *n *= 66), whether their disease was newly diagnosed (denoted by the cross symbol) or recurrent (denoted by the triangles), the mean number of PBMCs displaying p-STAT-3 was elevated to 11.8 ± 13.5%, which was significantly higher than that in healthy donors (*P *= 0.03) (Fig. 2). Among patients with recurrent GBM (denoted by the triangles) (*N *= 13; *n *= 24), the mean percentage of PBMCs displaying p-STAT-3 was 18.8 ± 17.1%, which was significantly higher than that in healthy donors (*P *= 0.0002). However, in newly diagnosed GBM patients (*N *= 15; *n *= 30) the mean p-STAT-3 level was 8.4 ± 8.8%, which was not significantly different from that of healthy donors (*P *= 0.3), although there was a trend toward increased levels in the GBM group.

Among grade III glioma patients (denoted by squares)(*N *= 10; *n *= 20; six patients were progressive from grade II and two were recurrent), the mean percentage of PBMCs displaying p-STAT-3 was 14.3 ± 9.4%, an elevation that was also statistically significant (*P *= 0.02) relative to healthy donor values. Because of insufficient patient numbers, no statistically meaningful conclusion can be drawn regarding differences in p-STAT-3 levels between newly diagnosed and recurrent grade III gliomas. Because of the referral pattern of patients to M. D. Anderson, insufficient sample numbers were obtained from patients with low-grade gliomas (denoted by stars), precluding a sufficiently powered conclusion; however, the low-grade glioma samples that were analyzed and also drawn during general anesthesia did not express p-STAT-3 levels above levels expressed in samples from healthy donors. Additionally, we did not detect elevations of the mean percentage of PBMC displaying p-STAT-3 (7.6 + 2.9%)(data not shown) in patients with a variety of metastasis to the CNS (n = 6; including four lung carcinomas, one bladder and one parotid gland), indicating that general anesthesia is not a contributing factor in the percent of PBMCs displaying p-STAT-3.

### Mean percentage of PBMCs displaying p-STAT-3 in patients whose GBM is without tumor progression is within the range of healthy donors

To determine if the mean percentage of PBMCs displaying p-STAT-3 continued to be elevated in GBM patients who had undergone gross total resection and whose disease appeared not to be progressing clinically or on magnetic resonance imaging (MRI)(denoted by circles), we obtained peripheral blood from these patients. The mean percentage of p-STAT-3 displaying PBMCs was 3.9 ± 3.5%, which was within the range of healthy donors (Fig. 2).

### Percentage of PBMCs displaying p-STAT-3 does not correlate with the percentage of p-STAT-3 positive tumor cells in the glioma

**Figure 3:**
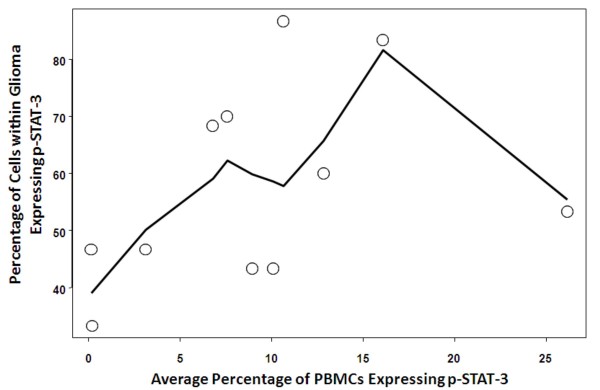
**Pair-wise scatter plots (with Loess smooth curves added) examining the relationship between tumor p-STAT-3 and PBMC p-STAT-3 expression**. The lack of a straight trend of the Loess curves indicates that there was not a correlation between tumor and PBMC expression of p-STAT-3.

To determine if the level of p-STAT-3 positive cells in the glioma correlated to the mean percent of PBMCs displaying p-STAT-3, we performed a subgroup analysis in which glioma specimens were stained with an antibody against p-STAT-3 and compared to the same patient's percentage of p-STAT-3 positive PBMCs. In pair-wise scatter plots with Loess smooth curves showing the relationship between the mean percentage of PBMCs displaying p-STAT-3 and the percentage of tumor cells displaying p-STAT-3, the Spearman correlation was 0.46 and a nonlinear trend indicated that there was no correlation between tumor and PBMC p-STAT-3 expression (*P *= 0.15) (Fig. 3 and Table 2). When excluding the outlier (N = 10), the Spearmen correlation is 0.51 (*P *= 0.13).

**Table 2:** Correlation of the percentage of PBMCs displaying p-STAT-3 compared to glioma expression

**Pathology**	**% of PBMCs displaying p-STAT-3**	**% of glioma cells displaying nuclear p-STAT-3**
Ganglioglioma	10.6 ± 0	87

Ganglioglioma	0.2 ± 0	33

Recurrent AA	12.8 ± 0.4	60

Newly diagnosed GBM	16.1 ± 0.4	83

Newly diagnosed GBM	0.1 ± 0	47

Newly diagnosed GBM	8.9 ± 0.4	43

Recurrent GBM	6.8 ± 0.4	68

Recurrent GBM	7.6 ± 0.2	70

Recurrent GBM	10.0 ± 0	43

Recurrent GBM	3.1 ± 0	47

Recurrent GBM	26.2 ± 23.7	53

### Percentage of Tregs in the CD4+ lymphocyte population does not correlate with amount of p-STAT-3 expression

**Figure 4:**
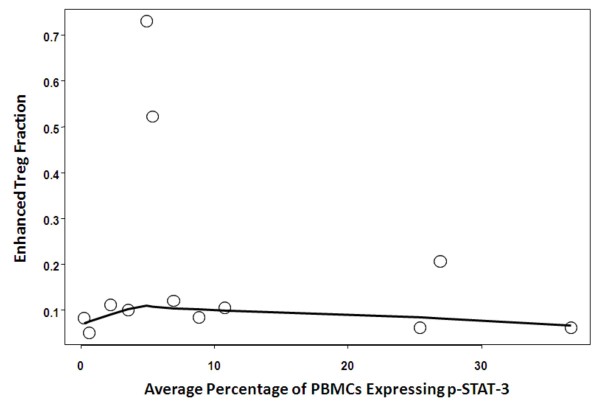
**Pair-wise scatter plots (with Loess smooth curves added) examining the relationship between PBMC p-STAT-3 and Treg fraction expression**. The lack of a straight trend of the Loess curves indicates that there was not a correlation between PBMC expression of p-STAT-3 and the induction of Tregs in malignant glioma patients.

To determine if the percentage of PBMCs displaying p-STAT-3 correlated with the degree of immune suppression as measured by the fraction of Tregs in the CD4+ compartment in glioma patients, we measured the percentage of FoxP3-positive Tregs in the CD4^+ ^lymphocyte population in a subset of GBM patients and compared the measurement to the same patient's percentage of p-STAT-3 positive PBMCs. In pair-wise scatter plots with Loess smooth curves examining the relationship between the mean percent of PBMCs displaying p-STAT-3 and the Treg fraction, the Spearman correlation was 0.03 and a nonlinear trend indicated that there was no correlation between the mean PBMC p-STAT-3 expression and an enhanced Treg fraction (*P *= 0.91) (Fig. 4). In healthy donors, the average fraction of FoxP3+ positive Tregs in the CD4^+ ^population was 10 ± 0.02% compared to 19 ± 21.0% in the GBM patient population, indicating that the Treg fraction was elevated in GBM patients with p value of 0.86

## Discussion

In this study, we found that a higher percentage of PBMCs expressed p-STAT-3 in glioma patients than in healthy donors. Compared to healthy donors, patients with anaplastic astrocytoma (WHO grade III) and recurrent GBM (WHO grade IV) had statistically significantly higher levels of the percent of PBMCs displaying p-STAT-3. Moreover, in patients with glioma that were without progression, p-STAT-3 levels were within the healthy donor range. These findings suggest that p-STAT-3 levels may be elevated in PBMCs when a tumor is present but not when there is no radiographic evidence of a tumor; however longitudinal data will be needed to correlate tumor progression and p-STAT-3 expression. One of the GBM patients whose MRI was questionable for tumor progression had radiation necrosis confirmed by biopsy; the mean percent of PBMCs displaying p-STAT-3 in this patient was 0.1%, suggesting an absence of tumor. This type of assay may be able to resolve the diagnostic dilemma of radiation necrosis versus tumor necrosis; however, this possibility will need to be validated in a separate study.

A limitation of this assay is that an increase in the mean percentage of PBMCs displaying p-STAT-3 was not detected in all cases of malignant gliomas; however, an elevation in this value during follow-up could alert the clinician that additional diagnostic testing may be indicated. We do not believe that steroids are a mitigating factor in the analysis of percentage of PBMCs displaying p-STAT-3 since all of the blood specimens from the surgical patients were obtained intraoperatively at which time all patients routinely receive dexamethasone. Within this group, were patients with both the highest and lowest percentage of PBMCs displaying p-STAT-3. We can't completely rule out a role for steroids in the percentage of PBMCs displaying p-STAT-3 since patients both receiving and not receiving steroids within the same histology could not be compared due to the routine administration of intraoperative dexamethasone. Furthermore, insufficient numbers of patients with lower grade tumors receiving similar doses of steroids precluded a statistically meaningful comparison to patients with malignant gliomas. The overall trend is supportive of a malignant glioma diagnosis playing a more meaningful role compared to steroids in elevated p-STAT-3 levels in this study; however, patients with other types of malignancies also have elevated PBMC p-STAT-3 expression [26] and the PBMC p-STAT-3 levels may be elevated in other medical conditions. Ultimately the percentage of PBMCs displaying p-STAT-3 will most likely be useful as a biomarker to monitor response to treatment to a variety of anti-STAT-3 agents, such as JSI-124 (cucurbitacin I) [38], WP1066 [26], arsenic trioxide [39-41], and antisense approaches [42], which are in various stages of preclinical and clinical trial testing.

We and others have shown that p-STAT-3 is upregulated in the vast majority of malignant gliomas [3,43]. It was somewhat surprising that the mean percentage of PBMCs displaying p-STAT-3 did not correlate with the number of tumor cells displaying p-STAT-3. However, many factors have been identified that induce p-STAT-3 expression, including growth factors and cytokines, such as IL-6 [44], elaborated by reactive astrocytes [45], epidermal growth factor [43], and Janus kinase 2 [46], and it is uncertain which of these, either individually or in combination, is the etiological agent for inducing p-STAT-3 in PBMC. Alternatively, there may be other yet-unidentified factors that induce p-STAT-3 in PBMCs. For example, Kortylewski et al. [47] recently showed that p-STAT-3 signaling in the tumor microenvironment induces IL-23, which is mainly produced by tumor-associated macrophages. Tumor-associated Tregs express the IL-23 receptor, which activates STAT-3 in this cell type, leading to upregulation of the Treg-specific transcription factor FoxP3 and the immunosuppressive cytokine IL-10 [47]. Perhaps it is the tumor expression of IL-23, IL-6, epidermal growth factor or Janus kinase 2 or an undefined factor that ultimately regulates the expression of PBMC p-STAT-3 levels, but this was not determined in our current study.

To ascertain if PBMC expression of p-STAT-3 correlated with the degree of immune suppression, we directed our attention specifically to the Treg fraction in the CD4 compartment since the Treg fraction is elevated in patients with malignant glioma patients [35]. Furthermore, we selected this particular marker of immune suppression because IL-2 has been shown to regulate FoxP3 expression in human CD4+CD25+ Tregs [48] by inducing STAT-3 binding of the first intron of the FoxP3 gene [20] and because STAT-3 inhibitors have been shown to inhibit Tregs [26,36]. However, we did not find a statistically significant correlation between PBMC expression of p-STAT-3 and an increase in the Treg fraction; this is similar to our previous finding of a lack of correlation between glioma-expressed p-STAT-3 and the presence of intratumoral Tregs [3]. Although p-STAT-3 may be a transcriptional factor related to induction of FoxP3 expression, it may not be the only factor that influences Treg generation. STAT-3 has been shown to be a potent regulator of many types of anti-inflammatory responses, including suppressing macrophage activation [22,23,27], natural killer cell and neutrophil cytotoxicity, and dendritic cell maturation and function [15]. Thus, secondary to the redundancy and multiplicity of immunosuppressive mechanisms that the p-STAT-3 pathway mediates, it was not entirely surprising that a single marker of immune suppression (i.e., Tregs) failed to correlate with PBMC expression of p-STAT-3.

## Conclusion

In summary, identifying diagnostic tests that alert us to the presence or recurrence of cancer in patients is advantageous for treatment and prognosis. An easy-to-perform, inexpensive blood test affords distinct advantages to the practicing neuro-oncologist, especially in the confounding conundrum of assessing treatment effects versus glioma progression. Ultimately, we have demonstrated that the percentage of PBMCs displaying p-STAT-3 may have utility as a clinical trial biomarker, but larger sample numbers will be required to validate the sensitivity and specificity of the assay.

## Abbreviations

APC: antigen presenting cell; CNS: central nervous system; FACS: fluorescence-activated cell sorter; FITC: Fluorescein isothiocyanate; FoxP3: forkhead box P3; GBM: glioblastoma multiforme; MRI: magnetic resonance imaging; *N*: sample size; *n*: number of measurements;PBMC: peripheral blood mononuclear cell; PBS: phosphate-buffered saline; PE: phycoerythrin; p-STAT-3: phosphorylated signal transducer and activator of transcription 3;STAT-3: signal transducer and activator of transcription 3; Treg: regulatory T cells

## Competing interests

The authors declare that they have no competing interests.

## Authors' contributions

WH, YW, CR-O, MKA-G, L-YK, RW, GNF and ABH contributed to the conception and design of the study. LMC, RW, GR, JW, SSP and ABH provided materials and patients for the study. WH, YW, CR-O, MKA-G, JW, L-YK, GNF, and ABH participated in data collection, and WH, YW, WQ, CR-O, JW, GFN and ABH participated in the data analysis and the interpretation of results. WH, YW and ABH contributed to the writing of the manuscript. All authors have approved the final version of the manuscript.
