# Correction: Detecting the percent of peripheral blood mononuclear cells displaying p-STAT-3 in malignant glioma patients

**DOI:** 10.1186/s12967-024-05093-y

**Published:** 2024-03-21

**Authors:** William Humphries, Yongtao Wang, Wei Qiao, Chantal Reina-Ortiz, Mohamed K. Abou-Ghazal, Lamonne M. Crutcher, Jun Wei, Ling-Yuan Kong, Raymond Sawaya, Ganesh Rao, Jeffrey Weinberg, Sujit S. Prabhu, Gregory N. Fuller, Amy B. Heimberger

**Affiliations:** 1 Department of Neurosurgery, The University of Texas M. D. Anderson Cancer Center, Houston, TX USA; 2 Department of Biostatistics, The University of Texas M. D. Anderson Cancer Center, Houston, TX USA; 3 Department of Pathology, The University of Texas M. D. Anderson Cancer Center, Houston, TX USA

**Correction: Journal of Translational Medicine (2009) 7:92** 10.1186/1479-5876-7-92

Following publication of the original article [1], we have been notified that there was a typo mistake in the title.

It is now:

yuDetecting the percent of peripheral blood mononuclear cells displaying p-STAT-3 in malignant glioma patients

It should be:

Detecting the percent of peripheral blood mononuclear cells displaying p-STAT-3 in malignant glioma patients

## References

[1] Humphries W, Wang Y, Qiao W, Reina-Ortiz C, Abou-Ghazal MK, Crutcher LM, Wei J, Kong L-Y, Sawaya R, Rao G, Weinberg J, Prabhu SS, Fuller GN, Heimberger AB (2009). Detecting the percent of peripheral blood mononuclear cells displaying p-STAT-3 in malignant glioma patients. J Transl Med.

